# Epigenetic Regulation of MicroRNA Genes and the Role of miR-34b in Cell Invasion and Motility in Human Melanoma

**DOI:** 10.1371/journal.pone.0024922

**Published:** 2011-09-19

**Authors:** Joseph Mazar, Divya Khaitan, Dan DeBlasio, Cuncong Zhong, Subramaniam S. Govindarajan, Sharmila Kopanathi, Shaojie Zhang, Animesh Ray, Ranjan J. Perera

**Affiliations:** 1 Sanford Burnham Medical Research Institute, Orlando, Florida, United States of America; 2 Department of Electrical Engineering and Computer Science, University of Central Florida, Orlando, Florida, United States of America; 3 Keck Graduate Institute, Claremont, California, United States of America; Rikagaku Kenkyūsho Brain Science Institute, Japan

## Abstract

Invasive melanoma is the most lethal form of skin cancer. The treatment of melanoma-derived cell lines with 5-aza-2′-deoxycytidine (5-Aza-dC) markedly increases the expression of several miRNAs, suggesting that the miRNA-encoding genes might be epigenetically regulated, either directly or indirectly, by DNA methylation. We have identified a group of epigenetically regulated miRNA genes in melanoma cells, and have confirmed that the upstream CpG island sequences of several such miRNA genes are hypermethylated in cell lines derived from different stages of melanoma, but not in melanocytes and keratinocytes. We used direct DNA bisulfite and immunoprecipitated DNA (Methyl-DIP) to identify changes in CpG island methylation in distinct melanoma patient samples classified as primary *in situ*, regional metastatic, and distant metastatic. Two melanoma cell lines (WM1552C and A375 derived from stage 3 and stage 4 human melanoma, respectively) were engineered to ectopically express one of the epigenetically modified miRNA: miR-34b. Expression of miR-34b reduced cell invasion and motility rates of both WM1552C and A375, suggesting that the enhanced cell invasiveness and motility observed in metastatic melanoma cells may be related to their reduced expression of miR-34b. Total RNA isolated from control or miR-34b-expressing WM1552C cells was subjected to deep sequencing to identify gene networks around miR-34b. We identified network modules that are potentially regulated by miR-34b, and which suggest a mechanism for the role of miR-34b in regulating normal cell motility and cytokinesis.

## Introduction

Melanoma, a cancer of pigment producing cells in the skin, has complex origins influenced by genetic predisposition, prolonged exposure to ultraviolet rays of the sun, and the extent of melanin pigmentation of the skin. As in most cancers, melanomas often have epigenetic changes in regulatory genes, such as CpG methylation in 5′-upstream *cis* regulatory elements, and chromatin protein modification signatures [1], [2], [3]. The recent discovery of miRNAs and their epigenetic regulation adds further complexity as they have been shown to be important influences on posttranscriptional gene regulation in cancer cells [4]. Since miRNA precursor genes are usually nested within other protein coding genes, often within intron sequences, misregulation of these protein-coding genes by epigenetic mechanisms may also be expected to cause aberrant regulation of the miRNA target genes. miRNA gene silencing by CpG island methylation has been reported in several cancers [5], [6], [7], though little is known in this regard for melanomas [8].

It has recently been reported that a number of miRNAs are differentially regulated in melanoma, several of which appear to regulate melanoma cell invasiveness [9], [10], [11], [12], [13], [14]. The miRNAs miR-221 and miR-222 down-regulate p27Kip1/CDKN1B and the c-KIT receptor mRNA levels, which leads to progression of neoplasia through enhanced proliferation and reduced differentiation of melanoma cells [15]. Another miRNA, miR-137, down-regulates the expression of MITF, a master regulator of cell growth, maturation, and pigmentation in melanoma cells [16]. We and others have shown that several miRNA genes are differentially regulated in melanoma cells, and one such miRNA, miR-211, is consistently reduced in melanoma but not in melanocytes [17], [18]. The epigenetic silencing of miR-375 has also been recently shown by us in melanoma [19], suggesting a mechanism for regulation of cellular morphology and tissue networking.

miR-34b was previously reported to be epigenetically regulated in several cancers, including melanoma, colorectal, and head and neck cancer [8], and reduces the oncogenic potential of a head and neck cancer-derived cell line [8]. The fact that epigenetic silencing of miR-34b was shown in all three cancers but played an inhibitory role in oncogenesis for only one suggests that this miRNA may play distinct roles in unrelated cancers. To our knowledge, the functional significance of miR-34b in normal melanocytes and melanoma cells has not yet been investigated. Here, we report that cell lines derived from malignant melanomas and melanoma patient samples have hypermethylated CpG islands in the 5′-upstream regions of several miRNA-coding genes, including that of miR-34b. We engineered two cell lines derived from metastatic melanoma to ectopically express miR-34b, and show that these cells exhibit reduced cell motility, decreased substrate attachment, and reduced invasion. Next-generation sequencing revealed additional potential target genes that are modulated by the ectopic expression of miR-34b in melanoma cells, and many are predicted to function in a network of interacting products enriched for cytoskeletal proteins. Collectively, these results suggest that epigenetic silencing of miR-34b may be responsible for some important oncogenic characteristics of melanoma cells, including cell motility and migration.

## Results

### Identification of epigenetically regulated miRNAs in melanoma cell lines

**Figure 1:**
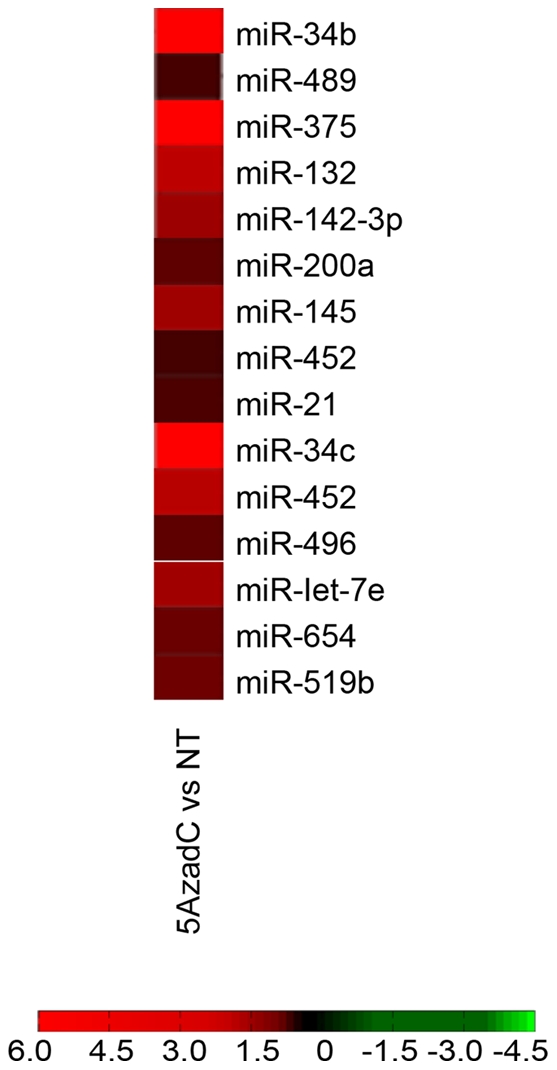
Activation of miRNA expression in response to 5-Aza-dC. Hierarchical clustering of differentially expressed miRNAs in melanoma cells after 5-Aza-dC treatment. 5-Aza-dC-induced expression changes ranged from highly upregulated (bright red) to marginally upregulated (dark red); black represents no change.

The reduced expression of genes that are under the control of CpG island methylation is often reversed by treating the cells with the DNA methyl transferase inhibitor 5-Aza-2′-deoxycytidine (5-Aza-dC). To assess the range and extent of miRNA expression under direct or indirect control of DNA methylation, we treated the melanoma cell line WM1552C (derived from a stage 3 malignant melanoma) with 5-Aza-dC and measured changes in miRNA gene expression using miRNA microarrays (see Methods). Several miRNAs, including miR-34b, -489, -375, -132, -142-3p, -200a, -145, -452, -21, -34c, -496, -let7e, -654, and -519b, were found to be up-regulated in WM1552C cells treated with 5-Aza-dC relative to untreated cells (Figure 1). Three miRNA genes (*mir-34b*, *-34c*, *-375*) whose products exhibited modulated expression in response to 5-Aza-dC treatment are known to contain CpG islands in their putative regulatory regions [20], [21]. Of these, *mir-34b* was found to be the most responsive to 5-Aza-dC treatment.

### CpG island methylation at the 5′ upstream region of *miR-34b* in melanoma

**Figure 2:**
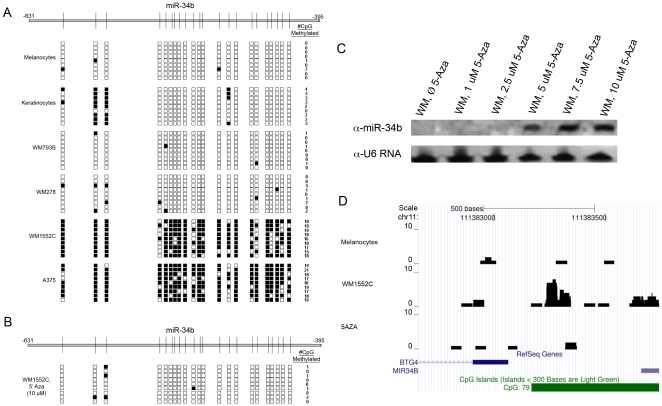
*mir-34b* 5′-UPS CpG island methylation in melanocytes, keratinocytes, and melanoma cells, and the effect of 5-Aza-dC demethylation on its expression in melanoma cells. A) Frequency of CpG island methylation in the *mir-34b* 5′-UPS measured by bisulfite genomic sequencing (horizontal line at top; numbers represent nucleotides relative to the transcription start site, and vertical lines represent the positions of CpG islands). Nine clones from each cell line were tested. Black boxes represent methylated sites, white boxes represent no methylation. WM793B = Stage 1, WM278 = Stage 2, WM1552C = Stage 3, A375 = Stage 4. B) WM1552C cells were treated with 5-Aza-dC and the putative *mir-34b* promoter in nine clones was then examined for CpG island methylation (conventions as described in A). C) Northern blot analysis of miR-34b expression in WM1552C cells after treatment with various concentrations of 5-Aza-dC. U6 RNA was used as a loading control. D) Methyl-DIP deep sequencing of the upstream CpG island sequences of *mir-34b* in melanocytes, WM1552C, and 5-Aza-dC treated WM1552C cells. Y-axes depict RPKM values and the horizontal green bar indicates the CpG island. The histograms represent the extent of CpG methylation.

*miR-34b* has a distinct CpG island located between –631 and –395 bp upstream of its precursor RNA start site, which contains 22 CpG dinucleotides. To examine the methylation patterns within the CpG islands, the genomic DNA of melanocytes and WM1552C cells was purified, bisulfite-treated, and sequenced. The sequence data were then compared to that of untreated genomic DNA isolated from the same cells. Negligible CpG methylation was observed in normal melanocyte and keratinocyte cell lines, and in melanoma cell lines obtained from stage 1 (WM793B) and stage 2 (WM278) melanoma tumors. However, this region was found to be highly methylated in cell lines obtained from stage 3 (WM1552C) and stage 4 (A375) melanoma tumors (Figure 2A). Treatment of WM1552C with 10 µM 5-Aza-dC largely erased CpG methylation at these sites (Figure 2B). miR-34b expression was increased in a dose-dependent manner by treating WM1552C cells with 5-Aza-dC, with maximum induction observed at 10 µM 5-Aza-dC (Figure 2C), the same concentration that reversed most of the upstream CpG methylation (Figure 2B). These results were confirmed in a parallel series of independent experiments in which methylated genomic DNA was first enriched by binding to reagents having high affinity to methylated DNA (see Methods), followed by next-generation DNA sequencing (Figure 2D).

### *miR-34b* CpG island methylation in melanoma patients and normal skin

**Figure 3:**
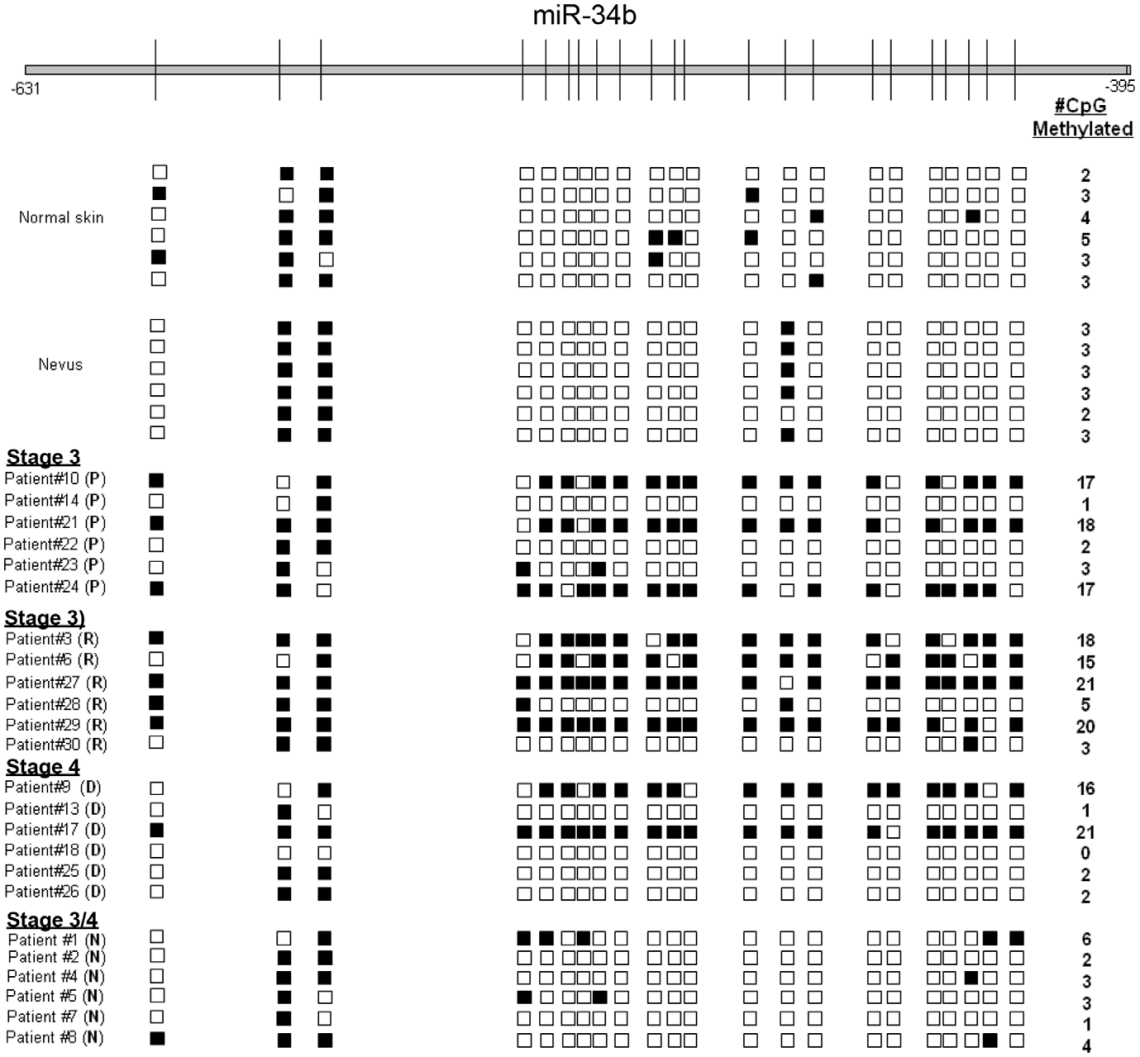
CpG islands 5′UPS of *mir-34b* are hypermethylated in clinical melanoma samples. CpG island methylation in the putative *mir-34b* promoter was measured by bisulfite genomic sequencing (conventions are as appeared in Figure 2). Six clones from each sample were tested. Patient samples are annotated as follows: (P) = primary melanoma, (R) = regional metastasis, (D) = distant metastasis, (N) = nodal metastasis.

Given the strong hypermethylation profile observed in the upstream sequences of miR-34b in late-stage melanoma cell lines, we next examined methylation levels in biopsied samples from patients with late-stage (Stage 3 and 4) melanoma. Bisulfite conversion and sequencing of DNA from normal skin and nevi samples revealed that the 5′ upstream sequences of *miR-34b* were largely hypomethylated (Figure 3), except at two sites located near the 5′ end (sites #2 and #3). This hypermethylation was also observed in keratinocytes (Figure 2A), suggesting that CpG methylation at these two sites may not be melanoma-specific.

We next examined CpG island methylation of *miR-34b* in 24 melanoma patient samples separated into four groups: (a) primary melanoma, (b) regional metastases, (c) distant metastases, and (d) nodal metastases. Three of six primary, four of six regional metastases, and two of six distant metastases melanoma samples were found to be hypermethylated (Figure 3). Interestingly, none of the nodal metastases showed significant methylation in this region.

The miR-34b methylation in melanoma patients indicates that methylation of this CpG island is detectable from patient samples, and may therefore hold the possibility of serving as a putative biomarker for stage 3 and 4 melanomas. However, there were only 24 patient samples of four groups of melanoma (primary, regional, distant and nodal). Thus, due to small sample size it is not possible to make an accurate statistical conclusion. Currently, we are continuing this study with a larger patient population with our collaborators (clinical partners). miR-34b expression in patient samples were tested by qRT-PCR and the results are shown as Figure S1.

### Ectopic expression of miR-34b in melanoma cells modulates both coding and non-coding RNA genes

To understand the significance of miR-34b in malignant melanoma, we stably expressed the *mir-34b* gene in the stage 3 melanoma cell line WM1552C, and analyzed genome-wide gene expression patterns in the engineered cells (WM1552C/34b) by deep sequencing (Figure S2). Total RNA was isolated from WM1552C/34b cells or cells expressing the vector only (WM1552C/VO) and the relative abundances of coding and non-coding RNAs were analyzed. Sequence reads were mapped to the latest version of the human genome sequence (hg19) using two mapping algorithms (see Methods).

The most differentially expressed genes were enriched for Gene Ontology categories of “cytoskeletal remodeling” (both TGF- and WNT-dependent and independent) and “cell adhesion” pathway network modules (Figure S3A), suggesting a direct or indirect mechanistic link between miR-34b expression and expression of genes related to cytoskeletal remodeling. Figure S3B and Table S1 show the most differentially expressed mRNAs, of which 34 were down-regulated and 19 up-regulated. Most of these genes are involved in regulating cell morphology, cell movement, and cell growth, and many of them (ALCAM, CAV1, DKK1, INHBA, MIA, MMP8, S100B, STC1, TGFBI, THBS2, TNS3, and WNT5A) have also been implicated in cell proliferation, migration, cell adhesion, invasion, and metastasis [22], [23], [24], [25], [26]. Interestingly, ALCAM, CAV1, DKK1, MIA, MMP8, S100B, and WNT5A have previously been shown to be involved in melanoma progression [27], [28], [29], [30], [31], [32], [33], and MIA and S100B are utilized clinically as biomarkers for melanoma [31], [32]. It is worth noting that CAV1, found to be down-regulated in WM1552C/34b cells, was previously identified as a target for miR-34b [34].

Next, we directed our attention to two of the differentially expressed mRNAs (THBS2 and DKK1) and validated their expression by Taqman qRT-PCR analysis (Figure S3C). THBS2, whose expression is up-regulated by ectopically expressed miR-34b, was chosen because it has previously been suggested to modulate cell adhesion and migration [35] and because it can act as a potent endogenous inhibitor of tumor growth and angiogenesis [36]. DKK1, whose expression is down-regulated by miR-34b, was chosen because its expression is also up-regulated in many cancers including myelomas, hepatocellular carcinomas, and breast and colorectal cancers [37], [38]. Additionally, high DKK1 expression correlates with poor prognosis in hepatocellular carcinoma [39]. qRT-PCR analysis revealed that THBS2 expression was 2.5-fold higher in WM1552C/34b cells than in WM1552C/VO cells, whereas DKK1 was nearly 10-fold lower in WM1552C/34b (Figure S3).

Next-generation RNA sequencing also revealed a number of miRNAs that were differentially expressed in response to miR-34b expression (Figure S4A, Table S2), and several such miRNAs (miR-20b, -134, -140, and -199b) are reportedly involved in cancer progression [40], [41], [42], [43]. In support of these findings, qRT-PCR analysis of miR-140 showed nearly 3-fold higher levels in WM1552C/34b cells than in WM1552C/VO cells (Figure S4B).

**Figure 4:**
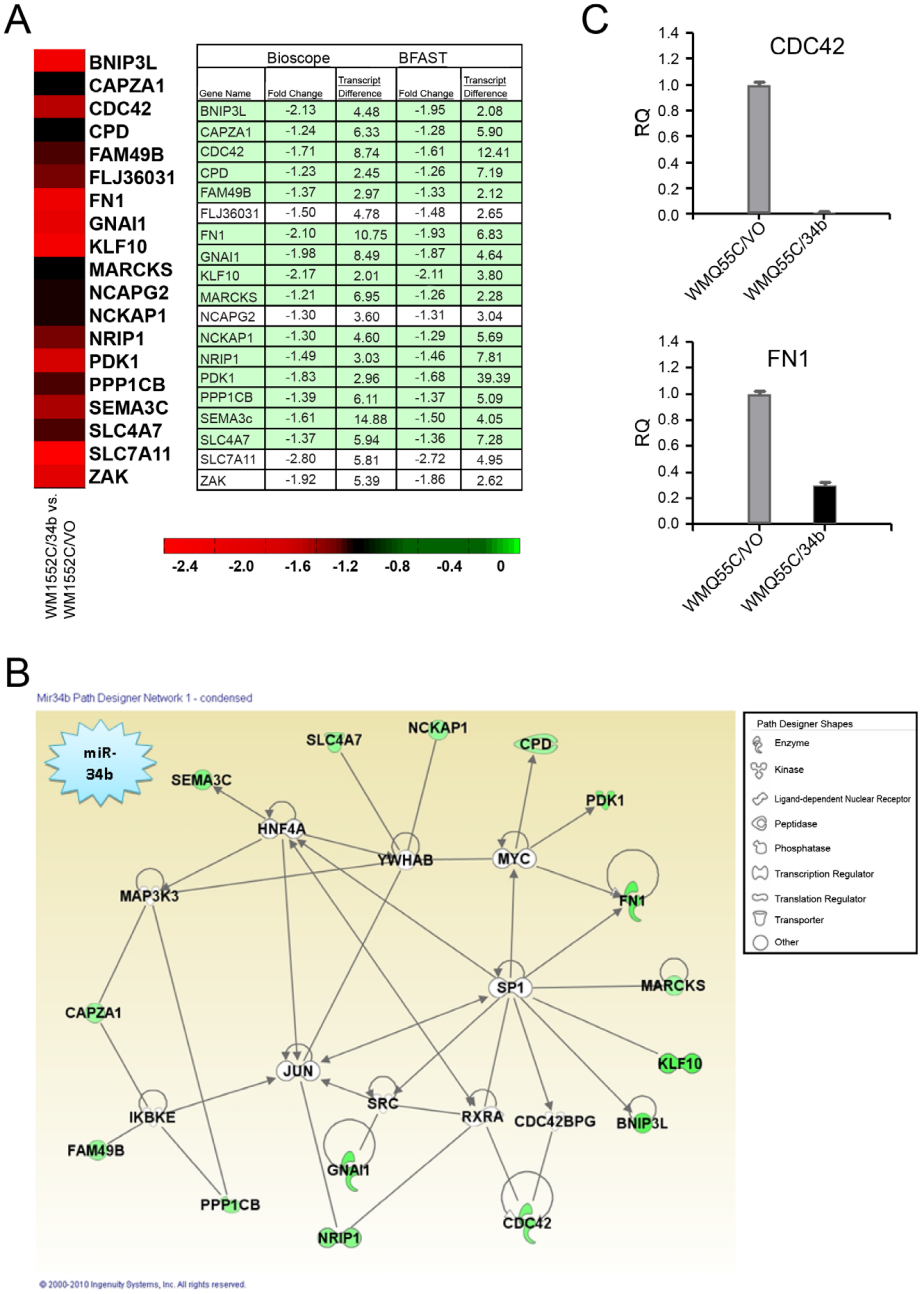
Differential expression of putative miR-34b target genes in miR-34b–expressing melanoma cells. A) A heat-map of the top potential miR-34b targets suggested by TargetScan 5.1, based on fold change and transcript differences between WM1552C/34b cells vs. WM1552C/VO cells. Green boxes indicate gene targets associated with the network of cytoskeletal rearrangement shown in (B). B) Pathway mapping of the top potential miR-34b targets reveals 15 targets all associated with the network of cytoskeletal rearrangement. Targets are indicated in green. C) qRT-PCR validation of the differential expression of putative miR-34b targets CDC42 and FN1 in vector-transfected or miR-34b-transfected WM1552C cells. Values are listed as relative quantities (RQ).

Deep sequencing revealed that mRNA expression of a group of 19 computationally predicted target genes were down-regulated in WM1552C/34b cells (Figure 4A). Of these putative targets, 15 are known to be involved in cytoskeletal rearrangement, cell morphology, cell adhesion, cell motility, and invasion (Figure 4B). Four of these 15 genes (CAPZA1, CDC42, NCKAP1, and PPP1CB) were previously shown to regulate actin filament polymerization and/or reorganization [44], [45], [46], [47]. We confirmed by qRT-PCR analysis that two of these genes (CDC42 and FN1) were down-regulated in WM1552C/34b cells compared with control cells (Figure 4C): CDC42 expression was 90% lower and FN1 expression was nearly 70% lower. Taken together, these data suggest that miR-34b regulates genes associated with cytoskeletal remodeling, migration, and invasion.

### The effect of miR-34b on melanoma cell growth, adhesion, migration, and invasion

**Figure 5:**
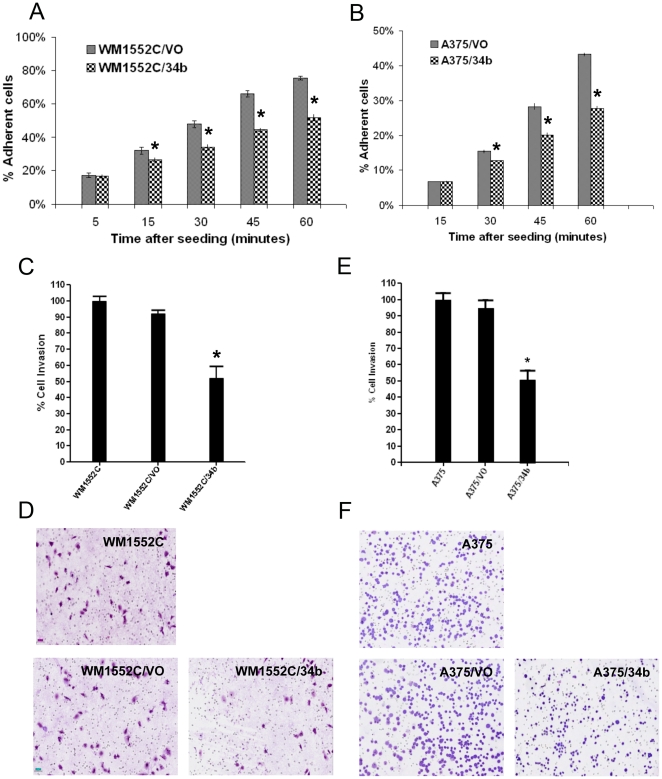
Effect of miR-34b on melanoma cell attachment and invasion. A) and B) Cell adhesion assays comparing WM1552C/VO and WM1552C/34b cells (A), or A375/VO and A375/34b cells (B). Data are percent of input cells bound, mean ± (SEM) of triplicates. C) and D) Cell invasion assays comparing untransfected WM1552C cells to stable WM1552C/VO and WM1552C/34b cells. E) and F) Cell invasion assays comparing untransfected A375 cells to stable A375/VO and A375/34b cells. All assays were performed in triplicate. Asterisks indicate statistical significance by Kruskal Wallis nonparametric test, *P*<0.005.

Since miR-34b expression appears to be epigenetically regulated in late-stage melanomas, we examined its effects on the functional phenotype of melanoma cells using various cell biology assays. WM1552C/34b cells showed decreased cell adhesion compared to WM1552C/VO cells, which was apparent within 15 minutes of plating and became more pronounced over the course of an hour (Figure 5A). Similarly, ectopic expression of miR-34b in A375 cell line decreased cell adhesion significantly compared with vector-transfected cells within 30 minutes of plating (Figure 5B).

We next examined the effect of miR-34b on the invasive properties of miR-34b-expressing cells. WM1552C/34b and A375/34b cells, as well as vector only and untransfected control cells, were seeded into invasion chambers and allowed to migrate for 48 hrs. There was a significant reduction (46%) in migration of WM1552C/34b cells compared with either WM1552C or WM1552C/VO cells (Figure 5C and 5D). A375/34b cells showed a similar trend (52%), reduction of cell migration compared to A375 or A375/VO cells (Figure 5E and 5F).

**Figure 6:**
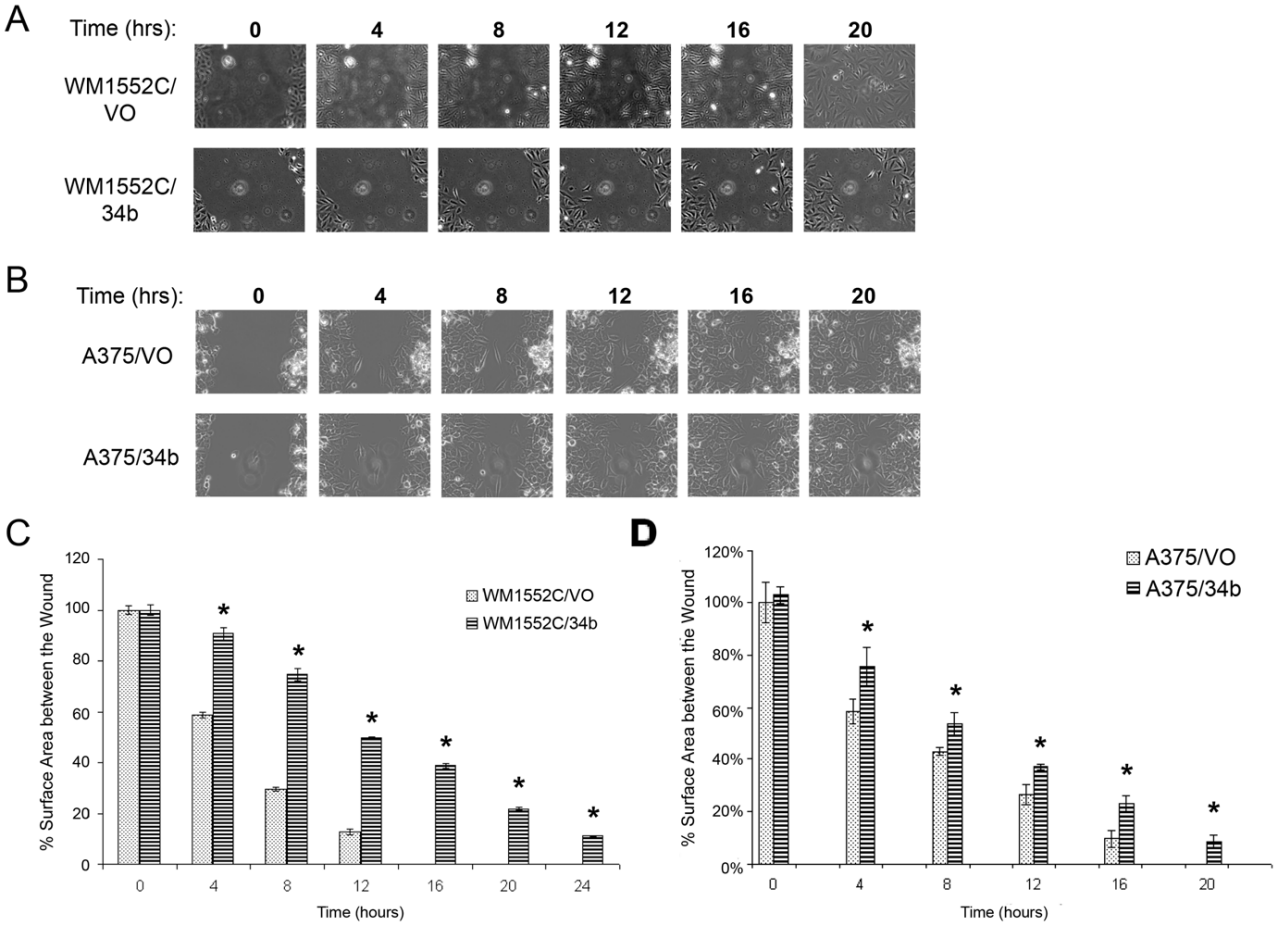
Effect of miR-34b on cell migration. Wound healing assay for melanoma cells. A) and B) Representative images of WM1552C/VO and WM1552C/34b cells or A375/VO and A375/34b cells. Images were taken between 0 and 20 hrs after scratch formation. C) and D) Quantitation of experiments shown in (A) and (B), performed in triplicate. The percent surface area between the wounds was calculated using NIS elements software. Each time point was compared with the 0 hr time point of the respective cell line. Asterisks indicate statistical significance by Kruskal Wallis nonparametric test, *P*<0.005.

Finally, we performed a wound-healing assay to assess the effect of miR-34b on cell motility. A striking difference was observed in the rates of motility of WM1552C/34b cells compared to control cells as early as 4 hrs after the initial scratch (Figure 6A and 6C). By 8 hrs, the WM1552C/34b-expressing cells had migrated less than half of the distance of WM1552C/VO cells, and full wound closure required 24 hrs for WM1552C/34b, compared with only 16 hrs for control cells. A less pronounced effect was seen in A375/34b cells, which closed the scratch within 24 hrs compared to 20 hrs for A375/VO cells (Figure 6B and 6D). Taken together, these results highlight the importance of miR-34b regulation for cell adhesion, invasion, and motility in human melanomas.

## Discussion

We have identified a group of epigenetically regulated miRNA genes in melanoma cells, including *miR-34b*, *-489, -375, -132, -142-3p, -200a, -145, -452, -21, -34c, -496, -let7e, -654, and -519b*. We used direct DNA bisulfite and immunoprecipitated methylated DNA (methyl-DIP) deep sequencing to confirm that the upstream CpG island sequences of one such miRNA gene (*miR-34b*) are hypermethylated in advance melanomas. By contrast, CpG methylation of miR-34b was less extensive in stage 1 and 2 melanoma samples, normal melanocytes, and keratinocytes. Abnormalities in CpG island methylation were also seen in melanoma patient samples classified as primary *in situ*, regional metastatic, and distant metastatic tumors. Interestingly, no miR-34b methylation was detected in nodal metastatic samples, but the significance of this observation remains unclear. It is possible that the panel of nodal metastatic melanoma samples (n = 6) was not large enough to detect hypermethylation. Alternatively, nodal metastatic melanoma cells might be epigenetically distinct from distant metastatic melanoma cells. Further studies are needed to resolve this issue.

Ectopic expression of miR-34b in the stage 3 and stage 4 melanoma cell lines caused a reduction in cell invasion, motility, and attachment rates, suggesting that the invasiveness of the WM1552C and A375 parental cell lines may be related to their low expression of miR-34b. RNA samples isolated from miR-34b-transfected and nontransfected WM1552C cells were subjected to deep sequencing to identify gene networks around miR-34b. These results identified network modules related to cytoskeletal remodeling and cell invasion, suggesting a mechanism by which miR-34b might regulate normal cell motility and cytokinesis. These observations are consistent with the existence of a global network of miRNAs and coding genes that is perturbed by miR-34b in melanoma cells; future analysis of this network may provide candidates for melanoma biomarkers.

Several groups have previously reported the importance of miR-34b in human melanomas [48], [49], [50], [51], [52], [53], [54]. Lujambio et al. [8], showed that the *miR-34b* upstream DNA sequences are hypermethylated in melanoma patient samples. Our results are consistent with this observation for advanced melanoma patient samples. Methylation-associated silencing of *miR-127* and *miR-124a* in T24 and HCT-116 cancer cell lines has been reported [6], [7]. Recently, epigenetic silencing of miR-375 was reported in melanoma cell lines and patient samples [19]. Interestingly, all three miR-34 members (a, b, and c) are reported to be methylated and silenced to varying degrees in many cancers [34], [53], [55]. Our methyl-DIP deep-sequencing results confirmed that the upstream CpG island sequences of miR-34b and miR-34c are highly hypermethylated in melanoma.

During melanoma formation, the initial genetic or epigenetic changes are thought to precede additional mutations and further epigenetic changes that affect the function of several signaling pathways. Aberrant DNA methylation patterns at the 5′ noncoding region of the *INK4a* gene was discovered in melanoma [56], which is consistent with the involvement of epigenetic factors in melanoma development or progression. Similarly, epigenetic silencing of PTEN expression occurs in certain malignant melanomas with no detectable mutation in the *PTEN* gene [57]. While the impact on melanoma development of epigenetic changes in several protein-coding genes is appreciated, there have been few reports of the impact of epigenetic regulation of non-coding RNAs, such as miRNAs.

The epigenetic modification of *miR-34b* may serve as a useful biomarker for early melanoma detection in humans, and therefore, one could propose to develop a novel sensitive *miR-34b* epigenetic biomarker assay to screen skin biopsies in melanoma patients. Including a panel of non-coding RNA epigenetic markers in to widely used pathological and genetic markers will be advantageous for both patients and pathologists. An investigation of *miR-34b* regulation and associated CpG island methylation in a large group of melanoma patient samples, in comparison with samples of matched normal tissues or melanocytic nevi, is both relevant and timely. Mir-34 group of miRNAs are known to be useful therapeutic target for various cancers [58], and MIRNATherapeutics Inc., (http://www.mirarx.com), a biopharmaceutical research company is currently focusing on *miR-34* group of genes as therapeutics.

In this study, we have identified many miRNA genes that are perturbed by treatment with a DNA methylation inhibitor, and have used deep sequencing to document a large collection of coding and non-coding RNA genes perturbed by the ectopic expression of miR-34b in melanoma cell lines. Thus, this report will also serve as a resource for future studies on the role of epigenetic regulation of non-coding RNAs in melanoma cells.

## Materials and Methods

### Cell lines and clinical samples

Experimental studies described in this manuscript used the human epidermal melanocyte cell line HEM-l (ScienCell, Catalog # 2200, grown in MelM media containing MelGS growth supplements, 0.5% fetal bovine serum (FBS), penicillin and streptomycin), human epidermal keratinocytes (HEK, ScienCell, Catalog # 2100, grown in Keratinocyte Medium, ScienCell, Catalog # 2101), and the melanoma cell lines WM793B (stage 1, Wistar Institute), WM278 (stage 2, Wistar Institute), WM1552C (stage 3, American Type Culture Collection Number: CRL-2808), and A375 (stage 4, American Type Culture Collection). Melanoma cells were grown in Complete Tu Medium containing a 4∶1 mixture of MCDB-153 medium with 1.5 g/L sodium bicarbonate and Leibovitz's L-15 medium with 2 mM L-glutamine, 2% FBS, and 1.68 mM CaCl_2_.

All clinical samples were graciously donated by Dr. James Goydos, Robert Wood Johnson Medical School.

### Isolation and bisulfite treatment of genomic DNA

Genomic DNA prepared from 10^7^ of each cell line. Cells were harvested by trypsinization, washed once in phosphate-buffered saline (PBS), and purified using the QiaAmp DNA mini kit (QIAGEN). DNA from 25 mg of patient samples was isolated by overnight incubation with proteinase K at 55°C, with subsequent purification using the QiaAmp DNA mini kit (QIAGEN). All samples were quantified using the ND-1000 spectrophotometer (Nanodrop). DNA (0.5 µg) was treated with sodium bisulfite using the EZ DNA methylation kit (Zymo Research) and eluted in 10 µL elution buffer.

### Sequencing of PCR products from bisulfite-converted genomic DNA for detection of CpG island methylation

Bisulfite-treated genomic eluate (2 µL) was used for bisulfite PCR using the following primer combinations: let7i f1 (GGGGGTAGTTTAGAATTAGTTGGTGTTTG) and let7i r1 (CCCCTTCTTTTCCTTTACCTTCCC) to produce a 301-bp product, 124a No-C-For (GGAAAGGGGAGAAGTGTGGG) and 124-3 Rev (CACCGCGTACCTTAATTATATAAAC) to produce a 260-bp product, & miR34b f1 (GAATTTGGGTTTTTATTTTTTAGG) and miR34b r1 (CCAAACCCTAAAACTAACTCTCTC) to produce a 236-bp product. PCR was performed using a 6-min hot start at 95°C, followed by 35 cycles at 94°C for 20 s, 54°C for 25 s, and 72°C for 30 s, ending with a 10-min extension at 72°C using AmpliTaq Gold (Applied Biosystems/Life Technologies). PCR products were gel purified using the QiaQuick gel extraction kit (QIAGEN) and cloned into the pCR4-TOPO vector (Invitrogen/Life Technologies). Nine clones for each miRNA candidate and 6 clones for each patient sample were sequenced using M13 primers and the BigDye terminator kit v1.1 (Applied Biosystems/Life Technologies), analyzed on a 3130×l Genetic Analyzer (Applied Biosystems/Life Technologies), and aligned using VectorNTi AlignX (Invitrogen/Life Technologies).

### Treatment of WM1552C cells with 5-aza-2′-deoxycytidine (5-Aza-dC)

WM1552C cells (5×10^5^) were plated into 75-cm^2^ flasks. Each flask was treated with 1, 2.5, 5, 7.5, or 10 µg/mL 5-Aza-dC or left untreated. Each day for 5 days, the cells were washed once with PBS, fed fresh medium, and treated as above. After day 5, the cells were trypsinized, washed once with PBS, and centrifuged at 1200 rpm for 5 min. Cell pellets were prepared for total RNA using the Trizol protocol (Invitrogen/Life Technologies), and RNA was quantified using the ND-1000 spectrophotometer (NanoDrop). The assay was performed in duplicate.

### TaqMan Low Density Arrays

For cDNA synthesis and real-time PCR by Taqman Low Density Array (TLDA), total RNA (800 ng) was subjected to 8 separate reverse transcription reactions (100 ng each) using Multiplex RT for TaqMan® MicroRNA Assays, Human Pool Set kit (Applied Biosystems/Life Technologies). The resulting cDNA (10 µL) was diluted 1∶62.5 with nuclease-free water. TLDAs consisting of a panel of 365 human miRNAs and 3 miRNA endogenous controls were run in triplicate for each sample. Diluted cDNA (50 µL) was added to 50 µL of 2× TaqMan Universal Master Mix (No AmpErase UNG; Applied Biosystems/Life Technologies). This 100 µL mixture was applied to the respective array port and the TLDA was then centrifuged twice and sealed. Quantitative real-time PCR was performed using the Applied Biosystems 7900 Real-Time PCR Sequence Detection System with the following thermal cycling parameters: 94.5°C for 30 s followed by 40 cycles of 97°C for 30 s, 59.7°C for 1 min.

### Quantitative Real-time PCR

Total RNA was isolated by the Trizol method (Invitrogen/Life Technologies) with subsequent quantification and integrity analysis performed using an Agilent 2100 Bioanalyzer (Agilent Technologies, Santa Clara, CA, USA). Total RNA (100 ng) was reverse transcribed using a High Capacity cDNA kit (Applied Biosystems/Life Technologies), and quantitative reverse-transcription PCR was carried out using TaqMan miRNA or mRNA Assays or SYBR Green mRNA Assays and a 7500 Real-Time PCR System (Applied Biosytems/Life Technologies) in accordance with the manufacturer's protocols. SYBR Green primers include CDC42 (CDC42 qPCR For – ctgcacctacccacatgcactcgt and CDC42 qPCR Rev - ttaactagtactgggagggggaaggg), FN1 (FN1 qPCR For – ggctgacagagaagattcccgagag and FN1 qPCR Rev - ccagtttagatggatcttggcagagagac), and THBS2 (THBS2 qPCR For – ttaccgcttcgtgcgctttgac and THBS2 qPCR Rev - aacagcgtgcccctggacttg). SDS1.2.3 software (Applied Biosystems/Life Technologies) was used for comparative Ct analysis, with RNU48, GAPDH, or β-actin serving as the endogenous controls.

### Northern blot analysis

Total RNA concentrated from each sample (20 ng from cell lines or 5-Aza-dC-treated melanoma cells as above), was analyzed by northern blot. Samples were separated in 15% TBE-urea polyacrylamide gels by electrophoresis and the RNA was electroblotted onto nylon membranes, cross-linked by ultraviolet light, prehybridized in Ultrahyb-Oligo (Ambion) for 30 min at 42°C, and hybridized with 5′-biotinylated anti-miRNA DNA oligonucleotides (100 nM each) at 42°C overnight. The blots were then washed, and the signal was detected by chemiluminiscence (Brightstar Detection kit, Ambion). Anti-U6 probes (10 pM) were used as a reference control.

### Construction of a melanoma cell lines stably expressing miR-34b

Oligonucleotides complimentary to the hsa-miR-34b genomic sequences were constructed (miR-34b pre For – gtgctcggtttgtaggcagtg and miR-34b pre Rev – gtgccttgttttgatggcagtg), containing *Hind*III and *Bam*HI sites on their respective 5′ and 3′ ends, then amplified from melanocyte genomic DNA (Amplitaq Gold, Applied Biosystems/Life Technologies). The product was then TOPO cloned into pCR4-TOPO (Invitrogen/Life Technologies). The vector construct was sequenced and the pre-hsa-miR-34b fragment was sub-cloned into pcDNA4/myc-HisA (Invitrogen/Life Technologies) using the *Hind*III and *Bam*HI sites to create pcDNA4/miR-34b. WM1552C and A375 melanoma cells (2.5×10^5^) were seeded into single wells of a 6-well plate and transfected with 5 µg pcDNA4/myc-HisA (Vector Control) or pcDNA4/miR-34b using Fugene 6 (Roche). The following morning, cells were selected with 600 µg/mL Zeocin for the following 15 days. The remaining stable cells were then expanded and named WM1552C/34b and WM1552C/VO (vector only) or A375/34b and A375/VO (vector only).

### Next-generation DNA sequencing and RNA-seq

Total RNA was isolated from samples using Trizol (Invitrogen/Life Technologies) and fragmented through RNase III digestion. SOLiD sequence protocols (Applied Biosytems/Life Technologies) require reverse transcription of these RNAs, priming with a ligated primer, and the resulting cDNA amplified and size-selected in a 6% urea gel with the help of SYBR Gold dye for sequencing. The sequence libraries (150–200 bp size fragments) were further amplified using a bead-based emulsion PCR optimized to physically isolate a single bead/cDNA molecule. This enables massively parallel amplification of monoclonal DNA species. For RNA-seq experiments, we deposited approximately 90 million beads per sample onto a glass slide and analyzed them using a ligation-based sequencing technology. Data were mapped to the human genome using both BioScope and BFAST software packages and analyzed at the Burnham Institute Bioinformatics core facility.

### Methylated DNA enrichment and library construction

Genomic DNA (melanocytes, WM1552C and WM1552C +5-Aza-dC) was fragmented to 50–400 bp (mean ∼250 bp) using a Covaris™ S2 System (Woburn, MA), and 10 µg was subjected to MBD-protein capture with the MethylMiner™ Methylated DNA Enrichment Kit (Life Technologies) following the recommended protocol. The methylated DNA was resuspended in 40 µL GibcoR UltraPure™ DNase/RNase-Free Distilled Water (Life Technologies) and quantified by UV absorbance spectroscopy. For single-fraction elution, buffer containing 2 M NaCl was used to elute methylated DNA captured using the MethylMiner™ kit. The methylated DNA fragments recovered were ethanol precipitated and resuspended. This DNA, and a sample of DNA that did not undergo enrichment with the MethylMiner™ kit (whole-genome, unenriched), were used for SOLiD™ System fragment library construction, which includes a gel-based size selection step to obtain a mean insert length of ∼150 bp.

### RNA-Seq gene analysis

Mapping of the SOLiD sequence data was performed using the standard pipeline of Blat-like Fast Accurate Search Tool (BFAST) [59] against the human genome (hg19) [60]. The BFAST algorithm first creates indices of the human genome using different masks (in this work, all 10 masks suggested in the BFAST manual were used). It then hashes the reads to a number of genomic locations based on the indices and performs detailed alignments between the reads and the genomic sequence at the hashed locations. The final output of BFAST contains the mapped genomic locations of each read and the qualities (represented via alignment scores) of the mappings.

The expression level of a gene can be calculated based on the mapping results. The BFAST algorithm successfully mapped ∼63% of the reads to the reference genome; among the mapped reads, ∼89% were uniquely mapped. Only uniquely mapped reads were considered to eliminate possible noise. For each gene (from the RefSeq annotation for hg19), a score was calculated as reads per kilobase of sequence per million reads (RPKM) [61].

The predefined mapping protocol for SOLiD sequencing on BioScope 1.0 was also used. BioScope was able to map ∼63% of the reads to the reference genome, ∼83% uniquely. The output was then fed into the Partek Genomics Suite for analysis. Again, an RPKM score was computed for each gene.

The results of the two analysis pipelines were compared to arrive at a consensus. Fold-change was computed for each gene, this was a ratio of the WM1552C/34b RPKM over the WM1552C/VO RPKM. For this calculation, a pseudo-count of 1.0 was used to overcome divide by zero errors and to normalize the data at low RPKM values. A list of genes was produced such that both programs agreed on the regulation direction (i.e. both upregulated or downregulated). The order of the genes was then directed by the corroborated fold change. For the open reading frames, a cutoff was established with a minimum fold change of +/−1.5 (1.5 for miRNAs, −1.2 for target genes). The transcript delta was also obtained; this was used to select genes for further testing, this cutoff was set at 10.0 for open reading frames (1.3 for miRNAs, −2.0 for target genes). The complete gene list is available in the supplemental data.

Of particular interest are the target genes of miR-34b. This target gene list was established from TargetScan 5.1 [62] utilizing the top 500 miR-34b targets, irrespective of conservation. The data acquired from deep sequencing was then filtered through the target list to arrive at a final putative target list.

### Cell adhesion assays

Trypsinized cells were counted, and 250,000 cells were seeded per well into 12-well plates. At 5, 15, 30, 45, and 60 minutes after seeding, floating cells were aspirated by rinsing the wells with PBS. The remaining cells in the wells were trypsinized, resuspended in cell media, and individually counted using the Countess® Cell Counter (Invitrogen/Life technologies). Each sample was assayed in triplicate at each time point, and each experiment was repeated twice.

### Invasion Assays

BD BioCoat™ growth factor reduced insert plates (Matrigel™ Invasion Chamber 12 well plates) were prepared by rehydrating the BD Matrigel™ matrix coating in the inserts with 0.5 ml of culture medium for 2 hrs at 37°C. The rehydration solution was carefully removed from the inserts, 0.75 mL Complete Tu medium containing chemoattractant (1% FBS) was added to the lower wells of the plate, and 0.5 mL of cell suspension (1×10^4^ cells, in serum-free medium) was added to each insert well. Invasion assay plates were incubated for 48 hrs at 37°C. Following incubation, the non-invading cells were removed by scrubbing the upper surface of the insert. The cells on the lower surface of the insert were stained with crystal violet and each trans-well membrane mounted on a microscopic slide for visualization and analysis. The slides were scanned using the Scanscope digital slide scanner, and the number of cells migrating was counted using Aperio software. Data are expressed as the percent invasion through the membrane, relative to the migration through the control membrane:

% invasion = Mean number of cells invading through the Matrigel insert membrane/Mean number of WM1552C (wild-type) cells migrating through membrane.

### In vitro wound healing assay

WM1552C/34b and WM1552C/VO cells or A375/VO and A375/34b cells were seeded on Mat Tek 1.5 mm tissue culture dishes until 90–95% confluent. Cell monolayers were then gently scratched with a pipette tip across the entire diameter of the dish and extensively rinsed with medium to remove all cellular debris. The surface area of the denuded surface was quantified immediately after wounding and again every 20 minutes for 24 hrs on the Nikon Bio Station IM. The extent of wound closure was determined by calculating the ratio of the surface area between the remaining wound edges for each time point to the surface area of the initial wound. These data were then expressed as the percentage of wound closure relative to the control conditions for each experiment. The surface area was calculated using NIS Elements software and performed in triplicate.

## Supporting Information

Figure S1 **miR-34b expression in patient samples.** miR-34b expression was analyzed by qRT-PCR on patient samples, and are expressed as RQ. Patient samples are annotated as follows: (Nev) = nevi, (NS) = normal skin (P) = primary melanoma, (R) = regional metastasis, (D) = distant metastasis, (N) = nodal metastasis.
(TIF)

Figure S2 **miR-34b expression in WM1552C/34b (stable ectopic expression), vector-only, cells treated with 5Aza-dC, or untransfected parental cells subjected to Deep Sequencing.** RNA expression profiles for miR-34b were measured in WM1552C melanoma cells which were stably transfected with either an expression vector containing the *mir-34b* gene (WM152C/34b), an empty vector (WM1552C/VO), no vector (WM1552C or “wild type”) or parental cells treated with 5-Aza-dC.
(TIF)

Figure S3 **Differential expression of mRNAs in miR-34b expressing melanoma cells.** RNA expression profiles were measured in WM1552C melanoma cells stably transfected with the *mir-34b* gene (WM152C/34b) or empty vector (WM1552C/VO). A) Global expression profiling using both Bioscope and BFAST revealed the three networks most perturbed in WM1552C/34b cells compared to WM1552C/VO cells (using GeneGo software). The expression ratios are transformed into logarithmic scale. B) Filtering of next generation data revealed the most up- or down-regulated coding genes in WM1552C/34b cells, shown as log fold change versus WM1552C/VO cells. C) qRT-PCR validation of the ORFs THBS2 and DKK1. Expression values are listed as RQ. Error bars are standard errors of mean.
(TIF)

Figure S4 **Differential expression of miRNAs in miR-34b-expressing melanoma cells.** RNA expression profiles were measured in WM1552C melanoma cells stably transfected with the *mir-34b* gene (WM152C/34b) or with empty vector WM1552C/VO). A) Filtering of next generation data revealed the most up-regulated miRNAs in WM1552C/34b cells, listed as log fold change versus WM1552C/VO cells. B) qRT-PCR validation of the miRNA miR-140. Expression values are listed as RQ. Error bars are standard errors of mean.
(TIF)

Table S1 **Differential expression of mRNAs in miR-34b-expressing melanoma cells.** The results of next generation data revealed the most up- or downregulated ORFs in WM1552C/34b cells, listed by fold change and transcript difference after consensus analysis by both Bioscope and BFAST (as compared to WM1552C/VO cells).
(PDF)

Table S2 **Differential expression of miRNAs in miR-34b expressing melanoma cells.** The results of next generation data revealed the most upregulated miRNAs in WM1552C/34b cells, listed by fold change and transcript difference after consensus analysis by both Bioscope and BFAST (as compared to WM1552C/VO cells).
(PDF)
