# Supplementary figures and images for: Epigenetic Regulation of MicroRNA Genes and the Role of miR-34b in Cell Invasion and Motility in Human Melanoma
Source: PLoS One. 2011 Sep 19;6(9):e24922. doi: 10.1371/journal.pone.0024922 (PMC3176288; doi:10.1371/journal.pone.0024922)

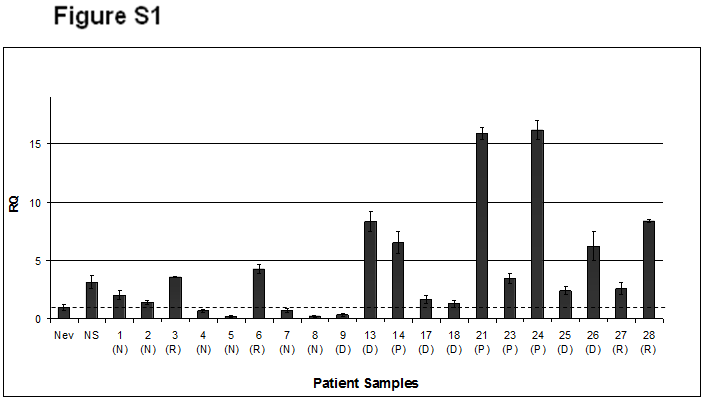

Supplement: Figure S1 — miR-34b expression in patient samples. miR-34b expression was analyzed by qRT-PCR on patient samples, and are expressed as RQ. Patient samples are annotated as follows: (Nev) = nevi, (NS) = normal skin (P) = primary melanoma, (R) = regional metastasis, (D) = distant metastasis, (N) = nodal metastasis. (TIF) [file pone.0024922.s001.tif]

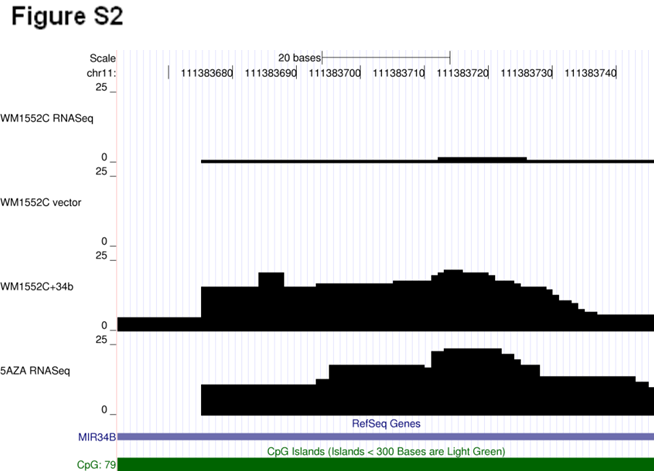

Supplement: Figure S2 — miR-34b expression in WM1552C/34b (stable ectopic expression), vector-only, cells treated with 5Aza-dC, or untransfected parental cells subjected to Deep Sequencing. RNA expression profiles for miR-34b were measured in WM1552C melanoma cells which were stably transfected with either an expression vector containing the mir-34b gene (WM152C/34b), an empty vector (WM1552C/VO), no vector (WM1552C or “wild type”) or parental cells treated with 5-Aza-dC. (TIF) [file pone.0024922.s002.tif]

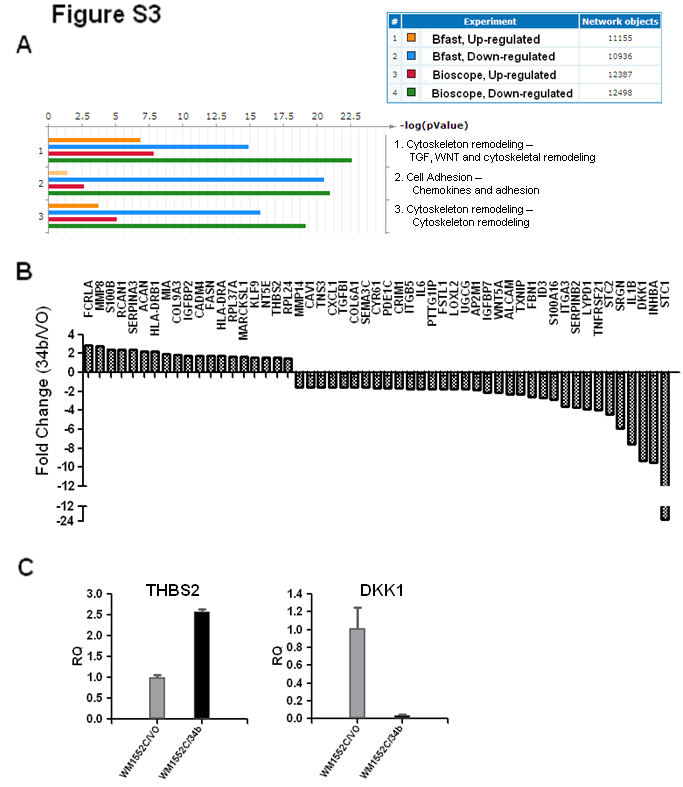

Supplement: Figure S3 — Differential expression of mRNAs in miR-34b expressing melanoma cells. RNA expression profiles were measured in WM1552C melanoma cells stably transfected with the mir-34b gene (WM152C/34b) or empty vector (WM1552C/VO). A) Global expression profiling using both Bioscope and BFAST revealed the three networks most perturbed in WM1552C/34b cells compared to WM1552C/VO cells (using GeneGo software). The expression ratios are transformed into logarithmic scale. B) Filtering of next generation data revealed the most up- or down-regulated coding genes in WM1552C/34b cells, shown as log fold change versus WM1552C/VO cells. C) qRT-PCR validation of the ORFs THBS2 and DKK1. Expression values are listed as RQ. Error bars are standard errors of mean. (TIF) [file pone.0024922.s003.tif]

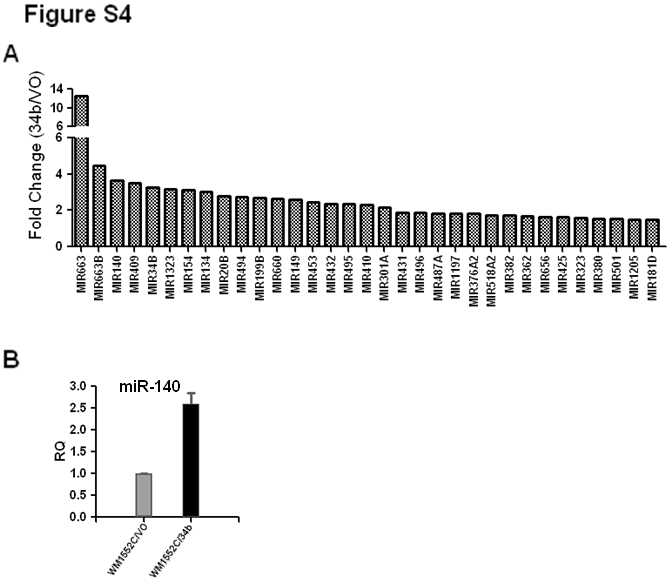

Supplement: Figure S4 — Differential expression of miRNAs in miR-34b-expressing melanoma cells. RNA expression profiles were measured in WM1552C melanoma cells stably transfected with the mir-34b gene (WM152C/34b) or with empty vector WM1552C/VO). A) Filtering of next generation data revealed the most up-regulated miRNAs in WM1552C/34b cells, listed as log fold change versus WM1552C/VO cells. B) qRT-PCR validation of the miRNA miR-140. Expression values are listed as RQ. Error bars are standard errors of mean. (TIF) [file pone.0024922.s004.tif]
