# Supplementary material for: Epigenetic Regulation of MicroRNA Genes and the Role of miR-34b in Cell Invasion and Motility in Human Melanoma
Source: PLoS One. 2011 Sep 19;6(9):e24922. doi: 10.1371/journal.pone.0024922 (PMC3176288; doi:10.1371/journal.pone.0024922)
Supplement: Table S2 — Differential expression of miRNAs in miR-34b expressing melanoma cells. The results of next generation data revealed the most upregulated miRNAs in WM1552C/34b cells, listed by fold change and transcript difference after consensus analysis by both Bioscope and BFAST (as compared to WM1552C/VO cells). (PDF) [file pone.0024922.s006.pdf]

Table S2. Differential expression of miRNAs in miR-34b expressing melanoma cells.

	Bioscope		BFAST	
miRNA Name	Fold Change	Transcript Difference	Fold Change	Transcript Difference
MIR663	12.50	70.74	1.71	29.24
MIR663B	4.48	35.55	1.99	27.73
MIR140	3.65	21.18	2.16	10.76
MIR409	3.50	12.15	5.11	7.38
MIR34B	3.26	11.50	4.95	3.95
MIR1323	3.16	9.85	1.58	2.73
MIR154	3.14	6.63	1.83	3.01
MIR134	3.02	6.50	1.80	1.42
MIR20B	2.78	6.06	1.50	5.24
MIR494	2.72	5.38	2.08	1.75
MIR199B	2.68	5.06	1.67	3.68
MIR660	2.65	4.70	3.11	5.07
MIR149	2.59	4.50	1.91	5.56
MIR453	2.44	4.29	2.93	2.27
MIR432	2.38	4.17	1.78	3.76
MIR495	2.35	4.14	2.94	3.47
MIR410	2.30	3.86	2.01	2.38
MIR301A	2.17	3.86	1.62	2.25
MIR431	1.90	3.63	1.75	3.13
MIR496	1.90	3.37	1.60	2.25
MIR487A	1.83	2.96	2.06	1.56
MIR1197	1.83	2.86	1.58	1.06
MIR376A2	1.82	2.78	2.08	1.14
MIR518A2	1.74	2.74	1.88	1.16
MIR382	1.72	2.70	1.95	1.12
MIR362	1.70	2.09	1.82	1.56
MIR656	1.64	2.08	1.97	1.08
MIR425	1.62	1.82	1.55	1.66
MIR323	1.57	1.76	2.24	1.78
MIR380	1.54	1.65	2.14	1.14
MIR501	1.52	1.44	2.26	1.26
MIR1205	1.51	1.32	1.72	1.06
MIR181D	1.51	1.32	1.55	1.22
